# Performance of ChatGPT in medical licensing examinations in countries worldwide: A systematic review and meta-analysis protocol

**DOI:** 10.1371/journal.pone.0312771

**Published:** 2024-10-28

**Authors:** Mingxin Liu, Tsuyoshi Okuhara, Xinyi Chang, Hiroko Okada, Takahiro Kiuchi

**Affiliations:** 1 Department of Health Communication, Graduate School of Medicine, The University of Tokyo, Tokyo, Japan; 2 Department of Health Communication, School of Public Health, Graduate School of Medicine, The University of Tokyo, Tokyo, Japan; 3 Department of Industrial Engineering and Economics, School of Engineering, Tokyo Institute of Technology, Tokyo, Japan; An Najah National University, STATE OF PALESTINE

## Abstract

**Introduction:**

In November 2022, the online artificial intelligence (AI) chatbot ChatGPT was released to the public, and swiftly garnered global attention because of its ability to provide detailed answers to complex queries. In medical field, ChatGPT has shown great potential to be used in medical education and has excelled in many English-language medical licensing examinations. However, due to the variability of medical licensing examinations in different countries, and ChatGPT’s particular proficiency in English, the previous literatures showed that ChatGPT is unable to pass medical licensing examinations from non-English-speaking countries or those not administered in English. To the best of our knowledge, this is the first study to review whether ChatGPT can demonstrate consistent accuracy across diverse medical licensing examinations and be used in medical education across countries.

**Objective:**

In this study protocol, we aimed to analyze and review the differences in performance of ChatGPT in medical exams in various language environments and countries, as well as its potential in medical education.

**Methods and analysis:**

A systematic review and meta-analysis was conducted using PubMed, Web of Science, and Scopus to collect papers testing the performance of ChatGPT in medical licensing examinations. We imported all the collected literatures into Rayyan and screened the literatures based on the selection criteria and exclusion criteria. The risk of bias and quality of included studies was assessed by using Mixed Methods Appraisal Tool (MMAT). Data from included studies was extracted into an Excel spreadsheet. All of the above processes were completed by two reviewers independently. A third reviewer was consulted in cases of disagreement. Finally, we provided both quantitative and qualitative analysis of the findings from the included studies.

**Trial registration:**

PROSPERO registration number: CRD42024506687.

## Introduction

### Background

In November 2022, the online artificial intelligence (AI) chatbot ChatGPT was released to the public, and swiftly garnered global attention because of its ability to provide detailed answers to complex queries [1]. Researchers have continued to explore whether ChatGPT can provide reliable and professional-level answers in different fields. One of these fields is medical licensing examination [2–18], which is a critical gateway in the medical education pathway and is used to assess the readiness of aspiring physicians in entering clinical practice [19]. These exams vary in format and content across different countries and typically test medical knowledge, clinical reasoning, and ethical decision making [20]. The advent of AI tools in this context is noteworthy because these tools can simulate complex human-like responses, thus potentially aiding in medical education and examination preparations [3]. The specific roles of ChatGPT in medical education and examination preparation are as follows: First, ChatGPT can serve as an effective knowledge resource. Although medical students can obtain medical knowledge via Google search, ChatGPT excels in presenting information in a concise and practical manner [21]. The responses from ChatGPT are highly logical and concordant, thus allowing medical students to easily understand the internal language, logic, and directionality of the relationships in the explanatory text [3]. Second, in medical licensing examinations that mainly consist of multiple-choice questions, students typically prepare by using third-party question banks (such as Uworld and Amboss) [21]. ChatGPT can act as a “virtual teaching assistant” by providing insights for each question and analyzing common mistakes to reinforce concepts in an interactive manner [22]. Third, ChatGPT has the ability to analyze images. Although this function is still in its early stages, it offers the potential for ChatGPT to serve as a “virtual tutor” that can analyze medical images (such as skin rashes and X-rays) [21]. Fourth, medical students face difficulty in allotting enough time for reviewing large amounts of information, practicing evidence-based medicine, and balancing clinical duties. ChatGPT can provide a simple summary of clinical trials and generate key practice points [21].

A study published in February 2023 was the first to test the performance of ChatGPT in the United States Medical Licensing Examination [2]. Subsequently, researchers have explored the performance of different versions of ChatGPT in medical licensing examinations from different countries and in different languages [2–18]. Regarding the research method, most researchers directly entered prepared medical licensing questions to ChatGPT and then collected and scored the responses of ChatGPT to evaluate its performance on the medical licensing examination and explore the potential possibilities of how ChatGPT could be used in medical education [2–18].

However, the application of ChatGPT in medical licensing examinations and medical education raises several important concerns. First, although the AI hallucinations of ChatGPT-4 have been greatly reduced compared with earlier versions, large language models in general still generate incorrect information because the data used to train these models are not always correct. Owing to the authoritative writing style of ChatGPT, it may be difficult for students to distinguish real knowledge from incorrect information [23]. Second, the accuracy of ChatGPT in handling diverse and complex subjects, which are common in medical exams, is critical [7, 18]. This includes not only factual medical knowledge but also the application of this knowledge to clinical problem solving and ethical dilemmas. Third, the ability of ChatGPT to adapt to various formats of medical licensing examinations across different countries, language environments, curricula, and assessment standards requires a thorough investigation.

Additionally, the use of AI in medical education also brings forth ethical and practical questions [22]. It challenges traditional educational paradigms and necessitates a reevaluation of what constitutes learning and assessment in a digitally augmented education landscape. The implications of relying on AI to train and educate medical professionals, who are responsible for human lives, warrant careful consideration.

Studies showed that there is significant variability in the performance of different versions of ChatGPT in medical licensing examinations in different countries and different language environments. For example, some studies showed that ChatGPT-3.5 passed the medical licensing examinations in the United States, Peru, and Iran [2, 11, 14] but failed in Japan and China [13, 17]. At the OpenAI DevDay conference held on November 6, 2023, OpenAI developers announced that the cut-off date of ChatGPT versions 3.5 and 4 were updated from September 2021 to January 2022 and April 2023, respectively [24]. Thus, we can assume that the performance level of ChatGPT in medical licensing examinations differed before and after the update.

To the best of our knowledge, only one systematic review has analyzed studies on the performance of ChatGPT-3.5 in English medical licensing examinations [25], and no study has reviewed the performance of ChatGPT in various language environments and countries. Therefore, the global perspective of the current review is crucial because medical education and licensure standards vary significantly across countries. This diversity offers a rich landscape for assessing the utility and adaptability of ChatGPT in different educational and cultural contexts. Additionally, this review will include studies on both ChatGPT versions 3.5 and 4.0 and will compare the performance of these different versions to provide a comprehensive review of the performance of ChatGPT in medical licensing examinations. Moreover, most of the studies we reviewed addressed the application of ChatGPT in medical education. We will integrate these viewpoints and provide comprehensive suggestions. The insights gained from this systematic review and meta-analysis will guide educators, policymakers, and technologists in harnessing AI effectively and ethically in medical education.

This systematic review and meta-analysis aim to bridge the gap in knowledge regarding the application of advanced AI tools, such as ChatGPT, in the domain of medical licensing examinations. By providing a comprehensive analysis of the performance of ChatGPT in various environments, this review seeks to contribute to the evolving discourse on AI in medical education and facilitate future developments and applications in this field.

### Study aims and objective

This study evaluates the medical knowledge accuracy of ChatGPT in diverse environments by reviewing its performance in medical licensing examinations across various languages and countries. It also explores the usability of ChatGPT in medical education and the potential issues that may arise.

Furthermore, given that physicians are responsible for patients’ lives and that their decisions are closely related to patient survival, it is irresponsible to hastily employ AI in medical education without a consensus on its accuracy and performance because this approach may lead to grave consequences. Therefore, there is an urgent need to review the performance of ChatGPT in various medical licensing examinations and assess whether ChatGPT can be used in medical education systematically.

This study aims to provide guidance on how to use ChatGPT in high-stakes and low-fault-tolerance fields, such as medicine; contribute to the discussions on the potential and challenges of integrating AI tools into medical education; and facilitate wise decision making on the use of technology in shaping future medical professionals.

The study objectives are as follows:

1. Performance Assessment: analyze and compare the performance of ChatGPT across different versions and languages with a focus on its accuracy, consistency, and relevance in answering medical-related queries and solving clinical scenarios.
2. Cross-Country Evaluation: explore the variability in the performance of ChatGPT across different countries by taking into account the diverse formats of medical licensing examinations and the specific medical curricula implemented in each country.
3. Utility and Adaptability: assess the utility of ChatGPT as a learning and revision tool for medical students preparing for licensing examinations, as well as its adaptability to the evolving content and structure of such exams.
4. Ethical and Practical Considerations: critically examine the ethical implications of employing AI in medical education and evaluation, particularly its effect on learning outcomes, student preparedness, and the integrity of medical licensing examinations.
5. Recommendations for Integration: provide recommendations on how ChatGPT and similar AI tools can be effectively and ethically integrated into medical education and licensing processes.

## Materials and methods

This systematic review protocol followed the Preferred Reporting Items for Systematic Review and Meta-Analysis Protocols, and the main study followed the Preferred Reporting Items for Systematic Reviews and Meta-Analysis flow diagram and guidance [26, 27]. The PRISMA-P checklist is available in S1 Checklist.

### Search strategy

We searched for specific query strings (Table 1) by using the advanced search function in PubMed, Web of Science, and Scopus and then exported the RIS files of all studies. Considering that ChatGPT was released in November 2022, we set the time period for collecting studies from November 2022 to June 2024. We then imported the RIS files exported from the three platforms into Rayyan [28]. The titles and abstracts of studies retrieved using the search strategy were screened independently by two authors (Mingxin Liu and Xinyi Chang) to identify studies that potentially meet the inclusion and exclusion criteria (Table 2). The full text of these studies were retrieved and independently assessed for eligibility by the two authors. Any disagreement between the two reviewers over the eligibility of particular studies was resolved via discussions with a third reviewer (Tsuyoshi Okuhara). The rationale for study exclusion was recorded as part of the screening process. This protocol was registered on the International Prospective Register of Systematic Reviews (PROSPERO) database on February 1st, 2024 (CRD42024506687).

**Table 1:** Query strings of WOS, Scopus and PubMed.

WOS	TS = (("ChatGPT" OR "GPT" OR "Generative pre-trained transformer" OR "AI" OR "artificial intelligence" OR "chatbot*" OR "large language model*" OR "LLM") AND ("medical licensing exam*" OR "medical education" OR "medical license" OR "licensing" OR "license" OR "exam" OR "exams" OR "examination"))
	OR
	TI = (("ChatGPT" OR "GPT" OR "Generative pre-trained transformer" OR "AI" OR "artificial intelligence" OR "chatbot*" OR "large language model*" OR "LLM") AND ("medical licensing exam*" OR "medical education" OR "medical license" OR "licensing" OR "license" OR "exam" OR "exams" OR "examination"))
	OR
	AB = (("ChatGPT" OR "GPT" OR "Generative pre-trained transformer" OR "AI" OR "artificial intelligence" OR "chatbot*" OR "large language model*" OR "LLM") AND ("medical licensing exam*" OR "medical education" OR "medical license" OR "licensing" OR "license" OR "exam" OR "exams" OR "examination"))
	OR
	AK = (("ChatGPT" OR "GPT" OR "Generative pre-trained transformer" OR "AI" OR "artificial intelligence" OR "chatbot*" OR "large language model*" OR "LLM") AND ("medical licensing exam*" OR "medical education" OR "medical license" OR "licensing" OR "license" OR "exam" OR "exams" OR "examination"))
Scopus	TITLE-ABS-KEY ((“ChatGPT” OR “GPT” OR “Generative pre-trained transformer” OR “AI” OR “artificial intelligence” OR “chatbot*” OR “large language model*” OR “LLM”) AND (“medical licensing exam*” OR “medical education” OR “medical license” OR “licensing” OR “license” OR “exam” OR “exams” OR “examination”))
PubMed	("ChatGPT" OR "GPT" OR "Generative pre-trained transformer" OR "AI" OR "artificial intelligence" OR "chatbot*" OR "large language model*" OR "LLM") AND ("medical licensing exam*" OR "medical education" OR "medical license" OR "licensing" OR "license" OR "exam" OR "exams" OR "examination")

**Table 2:** Inclusion and exclusion criteria.

Inclusion criteria	Exclusion criteria
The study tested the performance of ChatGPT in medical licensing examinations. Any type of literature. (peer-reviewed articles, conferences articles, preprints, letters, books, etc.) Literature published during November 2022 to June 2024. Literature on the performance of ChatGPT on medical licensing examinations written in all languages. Literature on any version of ChatGPT. Literature on multiple-choice questions, open-ended questions, and all other types of questions for medical licensing examinations. Literature published in all languages. In case the literature published in a language other than English, it will be translated into English using translation software.	Examinations other than comprehensive medical licensing examination (such as examinations for different medical specialties). Studies that are not related to ChatGPT. Duplicate studies.

### Data extraction and management

Data from the included studies were extracted into an Excel spreadsheet independently by two reviewers. The data were compared, and inconsistencies were resolved via consensus and/or by a third reviewer. The data to be extracted include the following: authors, year of publication, type of publication, country of the medical licensing examination, language in which ChatGPT was tested, duration of the test, the specific medical discipline in which the test was given, ChatGPT version, types of question entered into ChatGPT, performance and grades of ChatGPT, whether medical students participated in the comparisons, and whether there were discourses on medical education and on clinical and ethical issues.

### Assessing risk of bias in the included studies

The included studies discussed the performance of ChatGPT in medical licensing examinations and its potential in medical education. Given that the result contained both quantitative (score of ChatGPT responses) and qualitative (analysis of the content of the responses and discussions on the potential of ChatGPT in medical education) elements, the first and second reviewers (Mingxin Liu and Xinyi Chang, respectively) used Mixed Methods Appraisal Tool (MMAT) version 2018 for the independent quality assessment of the included studies [29]. A third reviewer (Tsuyoshi Okuhara) was consulted in cases of disagreement. MMAT consists of 27 questions, and 10 questions from parts 2 and 3 were excluded because they are not relevant to the included studies. The remaining 17 questions were used to assess quality. The results of the assessment using MMAT were presented in a concise table as a quality assessment of the included studies.

### Assessing risk of bias in this systematic review

To ensure the quality of this systematic review, the first reviewer (Mingxin Liu) assessed the risk of bias of this systematic review by using A Measurement Tool to Assess Systematic Reviews 2 (AMSTAR-2) [30], and the second reviewer (Xinyi Chang) double checked the appraisals. A third reviewer (Tsuyoshi Okuhara) was consulted in cases of disagreement. AMSTAR-2 is a critical appraisal tool for evaluating the methodological quality of systematic reviews. It consists of 16 questions that are designed to assess the methodological quality of systematic reviews, including the rationale for the review methods, comprehensive search strategy, and measurement of the risk of bias.

### Qualitative analysis

We provided a narrative synthesis of the findings from the included studies and structured the narrative synthesis by describing the studies according to the following issues:

1. Differences in the performance of ChatGPT for different types of questions (e.g., multiple-choice and open-ended questions).
2. Comparative analysis of the performance of ChatGPT across different medical specialty.
3. Performance comparison of ChatGPT in English and non-English question-and-answer environments.
4. Performance difference between ChatGPT and medical students in the same medical examinations.
5. Difficulties faced by ChatGPT in applying medical knowledge to clinical problems outside of medical knowledge and to possible ethical issues.
6. The role of ChatGPT in medical education and its potential.

### Quantitative analyses

We utilized the raw data for correct and total responses from each included study to determine the accuracy rate. The calculation rules were as follows: if a study employed a single set of questions for repeated testing, the accuracy rate will be the average score of all attempts, considering the total number of questions in the set. If the study tested both original language and translated English questions, the accuracy rate will be based on the scores from the original language questions. For studies that compared results with and without optimized prompts, the accuracy rate will be based on the scores without optimized prompts. In studies that included both multiple-choice and open-ended questions, the accuracy rate will exclude the scores from the open-ended questions. The accuracy rate was reported with 95% confidence intervals (CIs).

We conducted a meta-analysis of studies that tested ChatGPT using multiple-choice questions (MCQs) to address the following issues:

1. Performance comparison of ChatGPT version 3.5 and 4.
2. Performance comparison of ChatGPT in medical licensing examinations from English-speaking countries and non-English-speaking countries.
3. Performance comparison of ChatGPT before and after the update of the cut-off date on November 6, 2023

The I^2^ statistic was used to assess the effect of heterogeneity on the pooled results. When significant heterogeneity was present (I^2^ > 50%), a random effects model was used; otherwise, a fixed effects model was used. Accuracy was reported with a 95% confidence interval (CI). The significance level was set at p < 0.05. Meta-regression and subgroup analysis were conducted to examine the potential sources of heterogeneity and compare performances across different subgroups. A sensitivity analysis was conducted to assess the robustness of the meta-analysis results. The "metafor" and "meta" package in R 4.4.0 were utilized for the meta-analysis, publication bias and sensitivity analyses.

This study will summarize the performance of ChatGPT in different settings on the basis of the analysis of the above issues, discuss whether the current version of ChatGPT can be used in medical education, and provide targeted guidance on the use of ChatGPT in different settings and ethical issues that may be encountered.

## Discussion

To the best of our knowledge, this will be the first systematic review and meta-analysis to comprehensively review the performance of all versions of ChatGPT on medical licensing exams across different countries. Our protocol had clear definitions; established inclusion and exclusion criteria; and used a transparent and systematic approach for searching, screening, and reviewing studies. We extracted all data in standardized forms. We believe that the scope of our search was large enough and our inclusion criteria were broad enough to cover studies regarding the use of ChatGPT in different countries and languages for medical licensing examinations. By comprehensively evaluating the accuracy of medical knowledge possessed by ChatGPT and clarifying potential factors affecting ChatGPT performance, we have summarized the challenges and issues of applying ChatGPT to medical education and clinical diagnosis at this stage. The results of this study can guide educators, policy makers, and technologists in the effective and ethical use of AI in medical education. We believe that this systematic review is timely and will make a valuable contribution to fill the knowledge gap in the application of advanced AI tools, such as ChatGPT, in the field of medical licensing examination.

### Limitation

This systematic review excluded studies examining ChatGPT’s performance in different medical specialty exams, dental licensing exams, pharmacy exams, and other medical-related assessments. Future research should focus on evaluating ChatGPT’s performance in these specific medical domains.

Additionally, studies published in languages other than English, Japanese and Chinese were not considered in this review. This exclusion may result in the omission of literature assessing ChatGPT’s performance on medical licensing exams conducted in other languages.

## Supporting information

S1 Checklist PRISMA-P (Preferred Reporting Items for Systematic review and Meta-Analysis Protocols) 2015 checklist: Recommended items to address in a systematic review protocol*. (DOC)
